# Global Expression of Molecular Transporters in the Human Vaginal Tract: Implications for HIV Chemoprophylaxis

**DOI:** 10.1371/journal.pone.0077340

**Published:** 2013-10-15

**Authors:** Manjula Gunawardana, Madeline Mullen, John A. Moss, Richard B. Pyles, Rebecca J. Nusbaum, Jignesh Patel, Kathleen L. Vincent, Charles Wang, Chao Guo, Yate-Ching Yuan, Charles D. Warden, Marc M. Baum

**Affiliations:** 1 Department of Chemistry, Oak Crest Institute of Science, Pasadena, California, United States of America; 2 Departments of Pediatrics and Microbiology and Immunology, UTMB, Galveston, Texas, United States of America; 3 Human Pathophysiology and Translational Medicine Graduate Program, UTMB, Galveston, Texas, United States of America; 4 Center for Biomedical Engineering, University of Texas Medical Branch at Galveston, Galveston, Texas, United States of America; 5 Functional Genomics Core, Beckman Research Institute, City of Hope Comprehensive Cancer Center, Duarte, California, United States of America; 6 Bioinformatics Core, Department of Molecular Medicine, City of Hope National Medical Center, Duarte, California, United States of America; Rush University, United States of America

## Abstract

**Background:**

Pre-exposure chemoprophylaxis (PrECP) using antiretroviral agents is a promising strategy for the prevention of sexual HIV transmission in women. Molecular transporters in the human vaginal tract (VT) may play a pivotal role in determining drug disposition and, consequently, pharmacodynamic outcomes in these efforts. Little is known, however, on the expression of these transporters in vaginal tissues, representing a critical knowledge gap.

**Methodology/Principal Findings:**

Our study analyzed the genome-wide transcriptome in 44 vaginal tissue samples from 6 reproductive-age women undergoing gynecologic surgeries. The analysis revealed that, unexpectedly, a large number (43%) of gene isoforms corresponding to membrane transporters were over-expressed (above the median expression level) in all samples. A subset of 12 highly expressed membrane transporters was identified and contained 10 members (83%) of the solute carrier superfamily. The largest difference in membrane transporter gene expression was observed across subjects, but more subtle differential expression also was found along the anterior-posterior axis of the VT. Cross-validation of the microarray analyses with measurements RT-qPCR demonstrated high concordance between these data sets. Immunofluorescence labeling of membrane transporter proteins in vaginal tissues was highly dependent on tissue/cell types.

**Conclusions/Significance:**

Antiretroviral PrECP drugs currently under evaluation are substrates for molecular transporters that were commonly expressed, but fell into both over- or under-expressed categories in all samples, suggesting a complex role for carrier-mediated processes in determining the disposition of these xenobiotics in vaginal tissues. These findings hold important implications for the successful development of products, either oral or intravaginal, for female-controlled HIV PrECP.

## Introduction

As the HIV/AIDS pandemic enters its fourth decade, infection rates remain alarmingly high. The global incidence of HIV was estimated at 2.6 million in 2009, and 22 million more people are predicted to acquire HIV by 2031 [1,2]. These formidable statistics highlight the urgent need for effective antiretroviral pre-exposure chemoprophylaxis (PrECP) to prevent transmission in vulnerable populations. Systemic and topical PrECP using antiretroviral (ARV) agents is showing clinical promise for prevention of sexual HIV transmission [3-8], but there also have been a number of failed trials [2,9]. While the reasons for failure are unclear, it is undeniable that an appropriate drug disposition in key pharmacologic compartments is critical for a successful PrECP strategy [10-12].

Antiretroviral drugs have complex pharmacokinetic (PK) properties involving extensive drug metabolism, and transport by membrane-associated carrier proteins. Combination drug therapy often introduces drug-drug interactions that can result in toxic or sub-therapeutic drug concentrations and compromise treatment [13]. In addition, poor penetration of drugs into the intracellular compartment where HIV-1 replicates may contribute to the formation of virus sanctuary sites [14]. Molecular transporters from the ATP-binding cassette (ABC) and solute carrier (SLC) superfamilies are thought to play a central role in the disposition of ARV drugs [15-17]. Efflux systems can lead to a reduction of intracellular drug levels, decreasing antiviral activity and possibly promoting the development of resistant organisms [18]. Transporter-mediated absorptive processes may counter these effects [13]. Inhibition and induction of competing molecular transporters will lead to highly variable PKs among patients receiving PrECP, and the tissue-specific nature of transporter expression [13,19] introduces even more complexity. In the prevention of heterosexual HIV transmission in women, an understanding of types of molecular transporters present in the human vaginal tract (VT), and their interplay, is of critical importance. This area, however, remains largely unexplored [13].

Here, the global expression of membrane transporters in multiple locations of the VT of 6 women undergoing gynecologic surgery is described. A total of 44 tissue samples were studied by genome-wide transcriptome microarray analysis, and cross-validated with RT-qPCR measurements. Immunolocalization of membrane transporter proteins in these vaginal tissues also was carried out. The implications of these findings are discussed in terms of carrier-mediated drug disposition in HIV PrECP.

## Materials and Methods

### Subjects, vaginal tissue collection and processing

This study conformed to the principles of the Declaration of Helsinki. The study protocol was approved by the Institutional Review Board of the University of Texas Medical Branch at Galveston (IRB 12-233). The participants took part voluntarily and provided verbal informed consent prior to enrollment that included permission to use the samples obtained in future studies. Verbal consent was selected and approved *in lieu* of written consent to avoid placing undue burden on the subjects and further protect their confidentiality for this discarded materials study. The samples collected consisted of vaginal tissue collected during gynecologic surgeries, which are normally discarded. Samples and data were collected directly with a study ID, without any personal identifiers. A signed informed consent document would be the only link between the study ID and a subject name, therefore verbal consent, rather than written consent, was approved by the IRB. Verbal consent was documented for each participant on the study data collection sheet that was then stored in a locked file, accessible only to the study gynecologist, as required by the ethical review (Institutional Review Board).

Six nonpregnant, nonsmoking women between the ages of 20 and 56 years scheduled for vaginal surgery were recruited from the Galveston, TX metropolitan area during November and December 2012. Descriptive characteristics on the participants are provided in Table 1. At screening, participants were given information about the study and provided verbal informed consent. Criteria for inclusion required the subjects to be women, between 18-85 years of age, undergoing gynecologic surgery (hysterectomy or vaginal surgery) for clinical care purposes. Subjects were excluded if there was no plan for removal of tissue in the operating room.

**Table 1:** Subject characteristics.

	**Female (N = 6)**	
	**N**	**%**
**Age (years)**		
Mean ± SD	43 ± 14	
Range	20-56	
**Race**		
Black	2	33
White	4	67
Other	0	0
**Ethnicity**		
Hispanic	1	17
Non-Hispanic	5	83
**Body Mass Index**		
Mean ± SD	27 ± 6.6	
Range	16.9-35.2	
**Menopausal State**		
Pre-	3	50
Peri-	1	17
Post-	2	33

**Figure 1:**
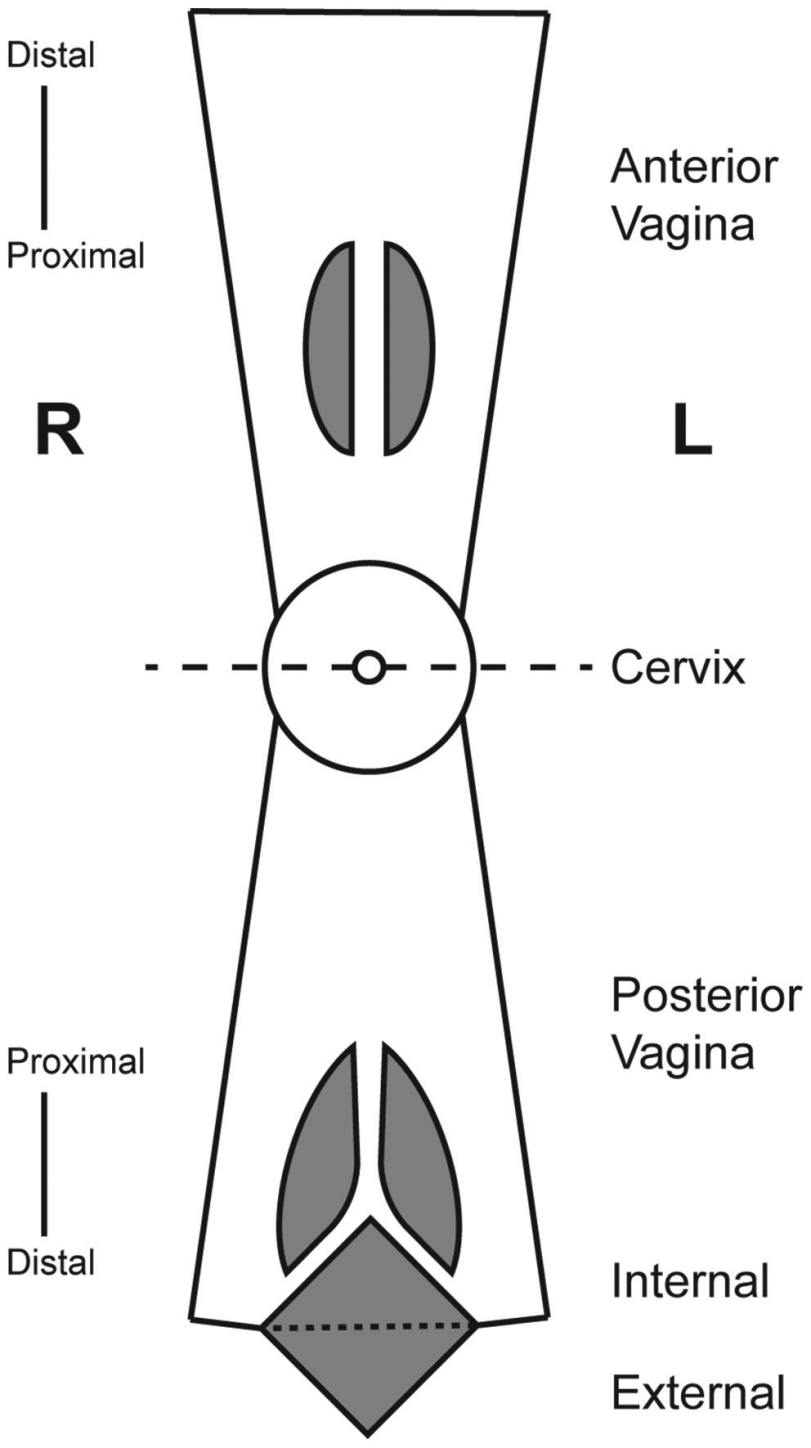
Diagrammatic representation of the vaginal tract identifying anatomical locations where tissue specimens were obtained. Dashed line through cervix indicates fold line and grey shaded areas represent surgical incisions. In this representation the cervicovaginal tract can be visualized as a sock-like tube, with the vaginal tract as the tube and the cervix as the toe. In the schematic, both sides of the tube are cut, opened anteriorly and posteriorly, and the anterior section folded over the cervix to afford the above visualization.

Vaginal tissues (Figure 1, Table S1 in Appendix S1) collected during surgery were placed immediately in sterile containers and processed. The specimens were dissected rapidly using sterile surgical blades and scissors to afford ca. 3 mm^2^ samples that were placed into prelabeled microfuge tubes containing 200 µL Aurum RNA lysis buffer (Bio-Rad Laboratories, Inc.) with supplemented β-mercaptoethanol (1% v/v). The samples were homogenized for 60 s with a clean, disposable pestle before freezing on dry ice. The time from tissue collection during surgery to processing typically was less than 20 min. A total of 44 tissue samples from different regions of the vaginal tract were processed. The samples were stored at -80°C.

### Extraction of total RNA

For total RNA extraction, samples were thawed on ice and mixed by repeat pipet disruption with 70% ethanol according to the procedure described in the technical protocol of the RNeasy Mini Kit (QIAGEN), which was used for subsequent purification according to the manufacturer instructions. This included an on-column DNase digestion with the RNase-free DNase kit (QIAGEN). The total RNAs were eluted once with 50 µL of RNase-free water. Initial RNA purity was assessed by diluting 4 µL samples with 146 µL of RNase-free water and measuring the OD 260 nm/280 nm ratio in a 96-well format using a SpectraMax® Plus Absorbance Microplate Reader (Molecular Devices). Samples were stored and transported at -80°C to the Functional Genomics Core at the City of Hope for further analysis.

### mRNA labeling, amplification and microarray hybridization, scanning

Gene expression profiles were measured using the Agilent one-color (Cy3 fluorochrome) microarray-based gene expression platform according to manufacturer's instructions. Total RNA quality was evaluated using the Agilent RNA 6000 Pico Kit with the Bioanalyzer 2100 (Agilent Technologies). Total RNAs (25 ng) were amplified and labeled using One-Color Low Input Quick Amp Labeling Kit (Agilent Technologies) in one run to minimize batch effects. Complementary RNA (cRNA) samples were hybridized onto SurePrint G3 Human Gene Expression 8x60K v2 arrays (G4851B, Design ID 039494, Agilent Technologies) for 17 h at 65°C in a rotator oven, followed by washing with Wash Buffers (Agilent Technologies). A randomized design was used to avoid biases. After washing, the slides were scanned using a Model G2505C Microarray Scanner (Agilent Technologies) and the hybridization signals were extracted using the Agilent Feature Extraction software, version 10.7.3.1.

### Microarray sample processing

Microarray gene expression values were calculated using the gProcessed Signal, which was normalized *via* log_2_ transformation and quantile normalization in Partek^®^ Genomics Suite™ (Ver. 6.6, Rev. 6.12.1227; Partek, Inc.). Subject samples were randomized across arrays so that batch effects would be independent of subject ID and sample location. Accordingly, the batch effect was removed for downstream analysis using the “Remove Batch Effect Function” (based upon a 3-way ANOVA between array ID, subject ID, and sample location). Over-expression analysis was performed using 44 samples. Differential expression analysis was performed using 41 samples (Table S1 in Appendix S1), with technical replicates removed to eliminate the possibility that the variation within groups would be artificially decreased. Microarray data have been submitted to the GEO repository (accession number: GSE49892).

### Over-expression analysis

A gene isoform based upon Agilent probe design was defined as “over-expressed” if normalized expression levels were above 3.5, and “under-expressed” if the levels were below 3.5. This is the median expression level for the sample distribution, which also corresponds to the change in slope in the sample histogram (Figure S7 in Appendix S1). All samples show identical signal distributions because the data were quantile-normalized. Counts and average expression values were calculated based upon over-expression status per sample (Dataset S1). Expression values were also averaged per subject and counts were calculated per subject using the same threshold for over-expression.

**Figure 2:**
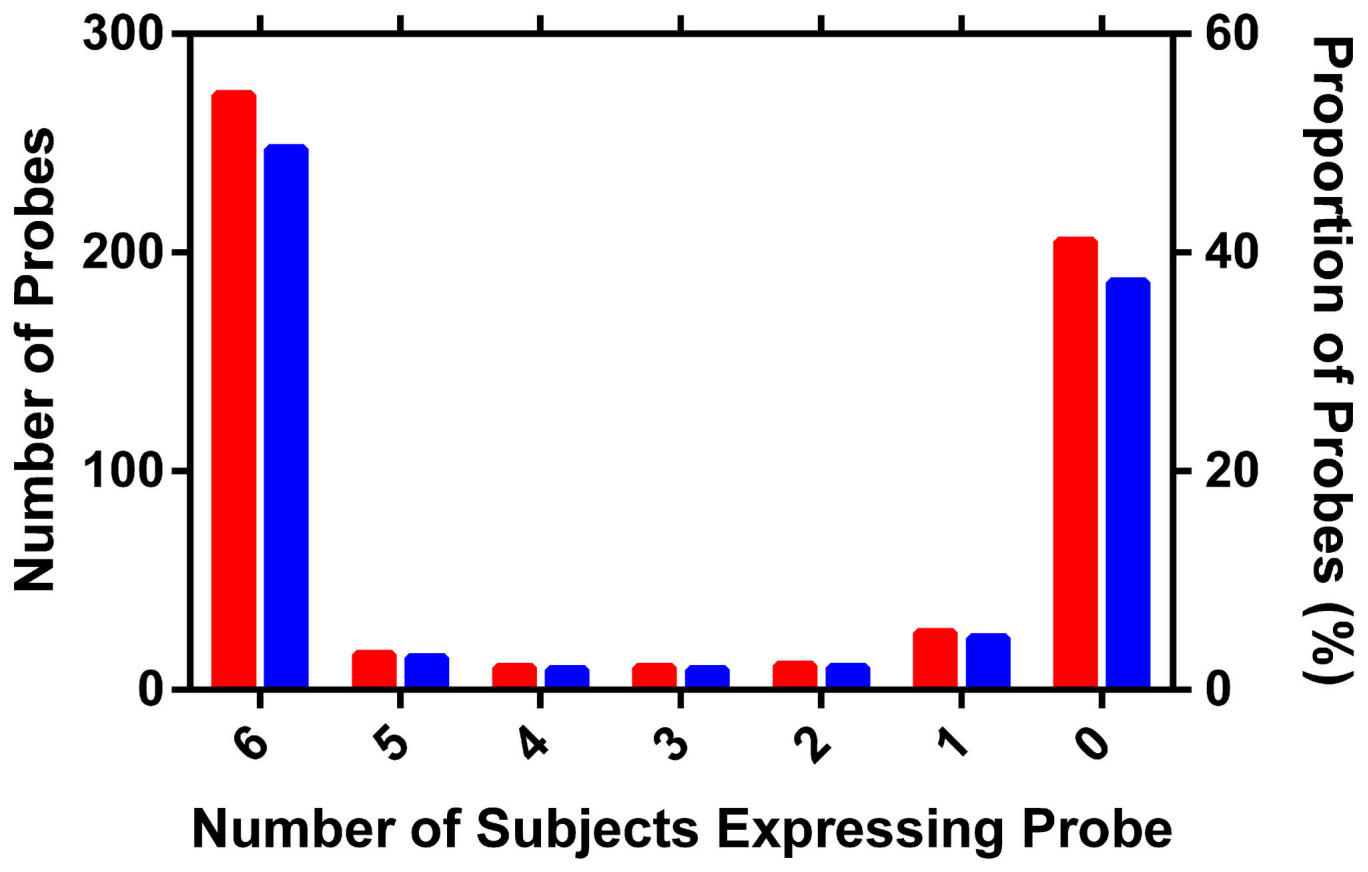
Histograms showing frequency of transporter expression per subject. Red bars, number of gene isoforms; blue bars, percentage of gene isoforms. Probes tend to be expressed in either all samples or no samples, and 50% of probes are expressed in all of the samples.

If a random variable is assumed to show above-median expression levels 50% of the time, then the observed proportion of high or low expression can be expressed as a proportion. Genes with above-median expression in all 6 subjects therefore would have an over-expression proportion of 100%. Using the prop.test function in R [20], genes with expression in either all 6 subjects or 0 subjects vary from a proportion of 0.5 with a *P*-value of 0.041 (Figure 2).

### Differential Expression Analysis

Partek^®^ Genomics Suite™ (Version 6.6, revision 6.12.1227; Partek, Inc.) was used to define differentially expressed genes. Fold-change values were calculated based upon the least-squares mean, per group. *P*-values calculated *via* 2-way ANOVA with appropriate linear contrast (e.g., anterior *versus* posterior; distal *versus* proximal; and left *versus* right). A 2-way ANOVA was used instead of a 1-way ANOVA because the signal varied strongly between subjects and the goal of the analysis was to identify changes in location independent of subject ID. False discovery rates (FDRs) were calculated using the method of Benjamini and Hochberg [21]. Genes showing strong differential expression were required to show a |fold-change| > 1.5 and an FDR < 0.05.

### Microarray gene expression cross-validation by RT-qPCR

Membrane transporter transcript expression profiling was carried out by reverse transcription-quantitative polymerase chain reaction (RT-qPCR) in a custom 96-well array, CAPH11899 (QIAGEN), containing 10 membrane transporter primer assays (*ABCB1*, *ABCC1*, *ABCC2*, *ABCC3*, *ABCC4*, *ABCG2*, *SLC15A1*, *SLC15A2*, *SLC22A6*, and *SLC22A8*) identified for cross-validation. The RT-qPCR array also contained primers for 3 housekeeping genes (*GAPDH*, *ACTB*, and *B2M*) as well as quality controls (genomic DNA control, positive PCR controls, reverse transcription controls). Total RNAs (25 ng µL^-1^, 8 µL, 200 ng) from the sample pool used in the microarray analysis corresponding to the above 41 vaginal tissue samples were converted into cDNA using the RT^2^ HT First Strand Kit (QIAGEN) according to the manufacturer’s instructions. The diluted cDNA solution (1 µL) then was added to RT^2^ SYBR Green qPCR Mastermix (2×, 12.5 µL, QIAGEN) and nuclease-free water (11.5 µL) to afford a final volume of 25 µL per reaction. The RT-qPCR was run in a Model CFX96 Touch™ Real-Time PCR Detection System (Bio-Rad Laboratories) using the following thermocycling parameters: 95°C for 1 min; 40 cycles consisting of 95°C for 15 s, 1.0°C s^-1^ ramp to 60°C for 1 min; and a melting curve consisting of 95°C for 10 s, 0.5°C s^-1^ ramp from 65-95°C. *C*_*t*_ values were calculated using the Bio-Rad CFX Manager software (Ver. 3.0) and a cutoff of 35 cycles was used to define detection. Two samples (OCIS-03 and OCIS-09) were excluded from further analysis on the basis of mean housekeeping gene *C*_*t*_ values above 26 cycles; the housekeeping gene *C*_*t*_ (mean ± standard deviation) of the remaining 39 samples was 22.07 ± 0.79 cycles. Δ*C*_*t*_ was calculated according to eq. 1:

$$\Delta C_{t}=C_{t}^{HK}-C_{t}^{MK}$$ (1)

Where *C*_*t*_^*HK*^ is the mean *C*_*t*_ for the three housekeeping gene transcripts in the same sample and *C*_*t*_^*MT*^ is the mean *C*_*t*_ for a given molecular transporter gene transcript.

Correlations were calculated per gene as well as for the combined set of all paired measurements. *P*-values were calculated using the cor.test function in R [20].

### Immunolocalization of selected molecular transporter proteins in paraffin-embedded, vaginal tissue sections

Full thickness human vaginal tissue specimens (ca. 3 mm^3^) from the different locations in the VT (Figure 1) collected during vaginal surgery as described above were immersion-fixed in zinc-buffered formalin (Z-fix, Anatech Ltd.) for at least 24 h at 4°C before embedding in paraffin. Thin sections (8 µm) from three depths of tissue were deparaffinized and rehydrated prior to autofluorescence quenching by incubation in 0.05% w/v Sudan Black B (199664-25G, Sigma-Aldrich) in 70% ethanol for 10 min at room temperature. Quenched sections were washed in deionized water for 10 min and then blocked with 5% non-fat milk in Tris-buffered saline for 30 min prior to incubation with the selected primary antibody for at least 16 h at 4°C. Primary antibodies targeting BCRP, MDR-1, MRP-1, MRP-2, MRP-3, MRP-4, OAT-1, and OAT-3 (Santa Cruz Biotechnology) were applied as a 1:50 dilution in 5% milk. After extensive washing, appropriate FITC-conjugated secondary antibody, diluted 1:500, was incubated for at least 2 h at room temperature in the dark. Secondary antibodies were: Donkey anti-Goat IgG (Southern Biotech), Goat and Mouse IgG and Goat and Rabbit IgG (KPL). Finally, sections were again washed extensively and covered with mounting media containing DAPI (Vector Labs), then a cover-slip was applied prior to visualization and imaging (Nikon Eclipse Ti, Nikon).

## Results

### Quality control of the total extracted RNA and the microarray results

RNA Integrity Number (RIN) measurements (7.1 ± 1.0, median ± standard deviation) on all 44 samples suggested that they were of sufficiently high quality for microarray analysis. Principal Component Analysis (PCA) of the microarray data showed that there are no outliers in the dataset (Figure S1 in Appendix S1) and qualified these data for further analysis. Both PCA (Figure S1 in Appendix S1) and hierarchical clustering (Figure S2 in Appendix S1) showed that subject ID was the dominant source of difference between samples. 

### Comparison of molecular transporter gene expression across all samples

The global expression of molecular transporters across 10 locations in the VT (Figure 1, Table S1 in Appendix S1) of 6 subjects with highly diverse characteristics (Table 1) was surveyed. Transporter gene over-expression patterns (see Material and Methods) were found to be highly consistent among subjects and sample locations (Figure 2). Membrane transporters were expressed less frequently than mitochondrial transporters (data not shown), but both types of transporters were expressed commonly: 46.1% of all transporter transcripts and 42.7% of membrane transporter transcripts, were over-expressed in 100% of the vaginal tract samples (*N* = 44).

The membrane transporters –determinants of drug disposition in active uptake processes- were ranked based upon averaged expression across all samples (Figure S3 in Appendix S1). An average threshold of 11 for highly expressed gene candidates was extrapolated from the distribution of averaged expression patterns. Only a small proportion of the samples exhibited averaged normalized expression above this threshold value (Table 2). Of the 12 most highly expressed membrane transporters, 10 (83%) were members of the SLC superfamily. Several (*SLC2A1*, *SLC16A3*, *SLC16A12*) are linked to lactate/pyruvate-dependent energy systems and are suspected to be active in the VT [22]. Analysis of these data with TiGER [23] predicted tissue-specific gene expression (Table S2 in Appendix S1). Only minor enrichment was predicted for these genes, as expected, because transporter genes typically do not show tissue-specific expression patterns (Figure S4 in Appendix S1). It is likely that the highly expressed membrane transporter genes identified here also are expressed in multiple tissue types throughout the body.

**Table 2:** List of most highly expressed membrane transporter genes across all samples.

**Symbol**	**Gene Name** [49]	**Primary Function** [50]
*RHCG*	Rh family, C glycoprotein	electroneutral and bidirectional NH_4_^+^ transporter. May regulate transepithelial NH_3_
*TAP1*	Transporter 1, ATP-binding cassette, sub-family B (MDR/TAP)	efflux of degraded cytosolic peptides across the endoplasmic reticulum into the membrane-bound compartment
*SLC2A1*	Solute carrier family 2 member 1	Glucose transporter
*SLC6A9*	Solute carrier family 6 member 9	High affinity, Na^+^- and Cl^-^-dependent neurotransmitter transporter
*SLC16A3*	Solute carrier family 16, member 3	H^+^-linked monocarboxylate transporter that catalyzes the rapid transport of numerous monocarboxylates (e.g., lactate, pyruvate, , β-hydroxybutyrate and acetate) across the plasma membrane
*SLC16A12*	Solute carrier family 16, member 12	See above
*SLC22A18*	Solute carrier family 22, member 18	Transporter of organic cations based on an H^+^ efflux antiport mechanism
*SLC24A3*	Solute carrier family 24, member 3	Plasma membrane Na^+^/Ca^2+^ exchanger
*SLC35E1*	Solute carrier family 35, member E1	Integral membrane transporter with unknown function
*SLC35E4*	Solute carrier family 35, member E4	See above
*SLC38A10*	Solute carrier family 38, member 10	Putative Na^+^-dependent amino acid/H^+^ antiporter
*SLC44A1*	Solute carrier family 44, member 1	Choline transporter

### Subject effect on molecular transporter gene expression

**Figure 3:**
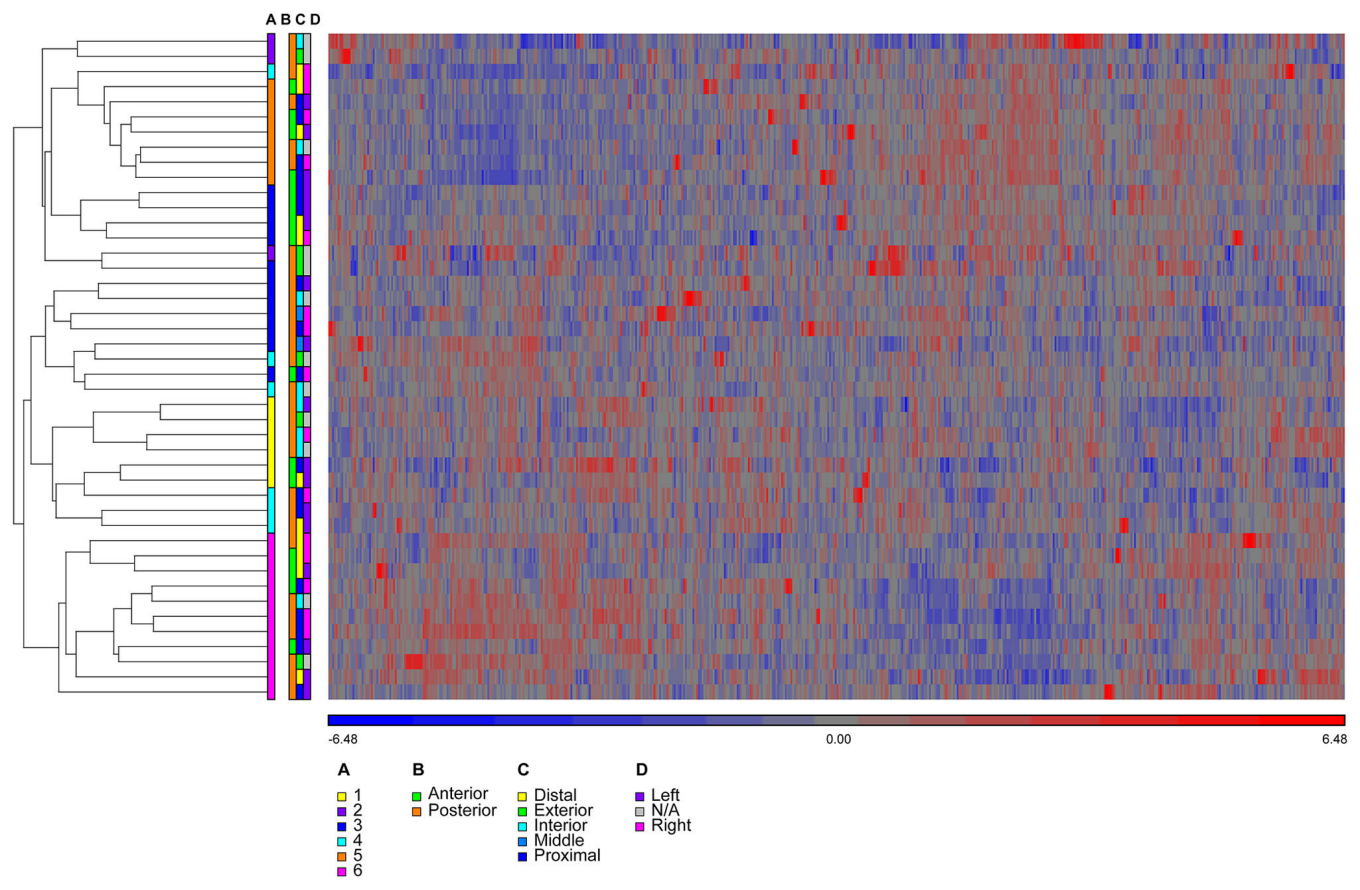
Clustering of membrane transporters across all samples from 6 subjects. (**A**) Color bar indicates strong clustering among samples from the same subject, for subjects 1-6. Color bars defining clustering along the following axes in the vaginal tract (Figure 1) show no discernible pattern: (**B**) anterior-posterior, (**C**) distal-proximal, and (**D**) left-right. The heatmap shows clustering of the standardized expression values, where probes with high expression are shown in red and probes with low expression are shown in blue (see color key below the heatmap).

**Figure 4:**
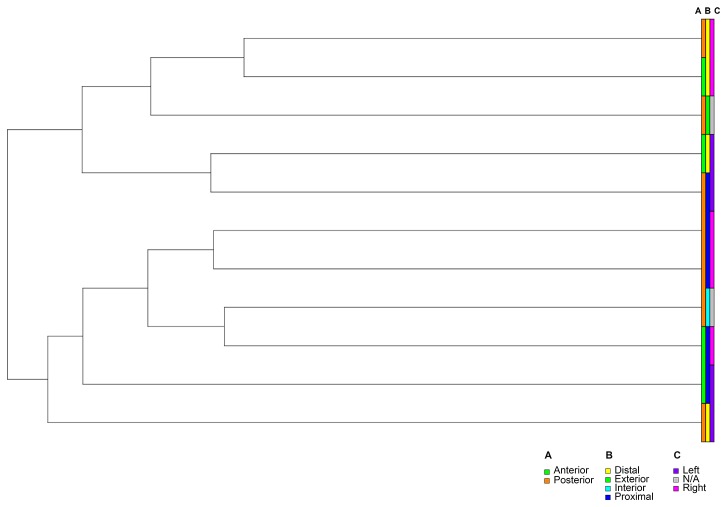
gram showing hierarchical clustering of genome-wide transcriptome in vaginal samples obtained from Subject 6. Color bars defining clustering for all probes along the principal axes in the vaginal tract (Fig. 1) show no strong pattern: **(A)** anterior-posterior, **(B)** distal-proximal, and **(C)** left-right.

**Figure 5:**
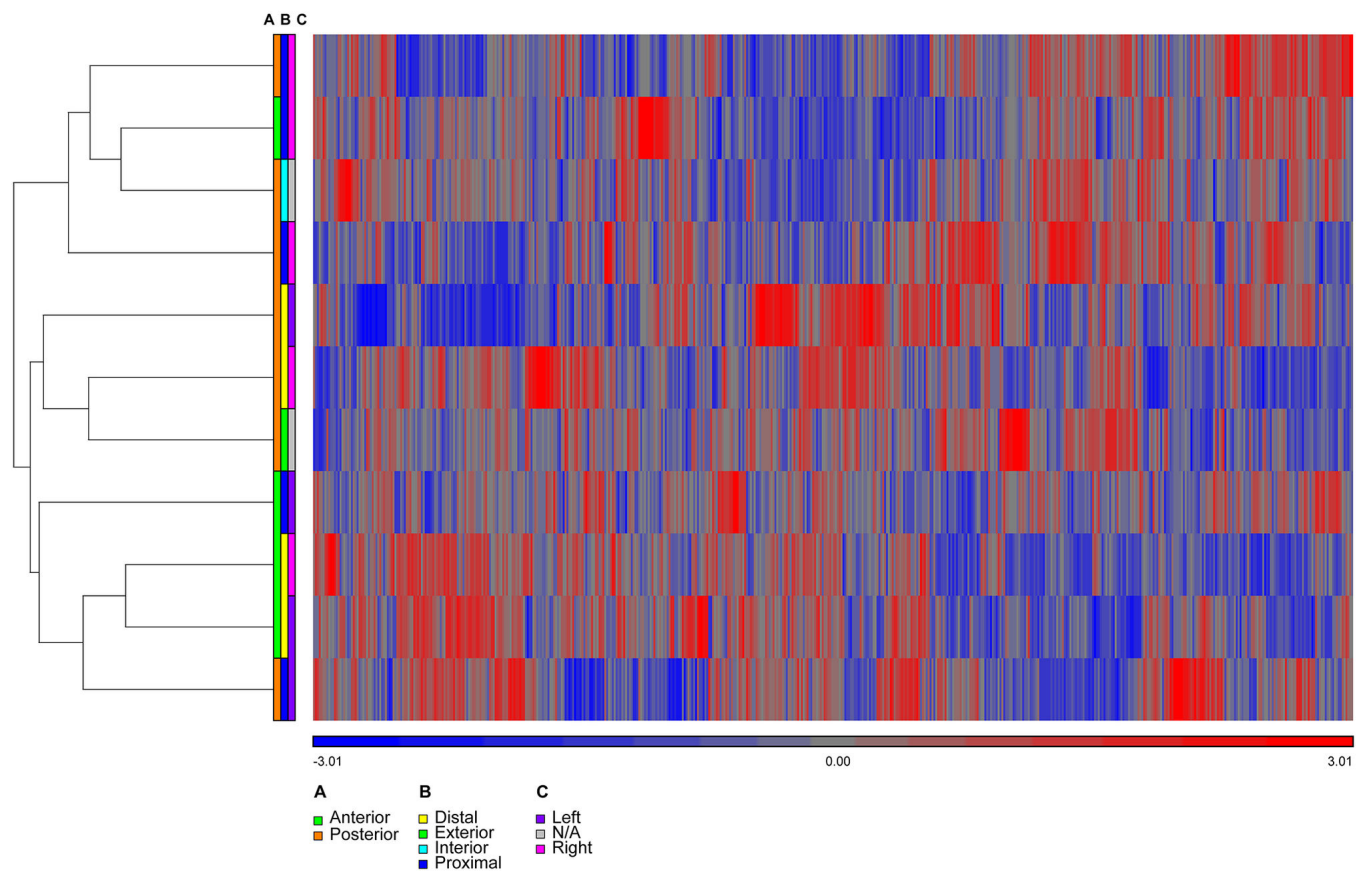
Membrane transporter transcript clustering of samples obtained from Subject 6. The clustering for all probes (i.e., transcriptome, Figure 4) and the membrane transporters (this figure) is approximately the same. Color bars defining clustering for all probes along the principal axes in the vaginal tract (Figure 1) show no strong pattern: (**A**) anterior-posterior, (**B**) distal-proximal, and (**C**) left-right. The heatmap shows clustering of the standardized expression values, where probes with high expression are shown in red and probes with low expression are shown in blue (see color key below the heatmap).

The clustering of membrane transporters was qualitatively similar to clustering of genome-wide expression values (Figure 3 *versus* Figure S2 in Appendix S1), strongly suggesting that the principal factor determining gene expression levels was the donor, an observation that was supported by F-statistics calculated across all genes on the microarray (Figure S6 in Appendix S1). Likewise, clustering for Subject 6 –the subject with one of the largest number of representative sample locations (Table S1 in Appendix S1)- showed qualitatively similar expression patterns among sample locations (Figures 4, 5). The minor differences in clustering for transporter genes probably were due to increased random variation in a smaller sample size. Comparing clustering across all samples to that within Subject 6 demonstrated that expression differences between sample locations were much weaker than expression differences between subjects.

### Differential membrane transporter gene expression

Although the subject ID clearly was the dominant factor associated with the gene expression changes measured here, it was of interest to determine if significant gene expression changes could be detected among any of the three dimensions of the vaginal tract: anterior *versus* posterior, distal *versus* proximal, and left *versus* right (Figure 1 and Figure S6 in Appendix S1). The analysis identified six membrane transporter genes that met our criteria for differential expression and exhibited stronger expression differences across the anterior-posterior axis (Table 3 and Figure S5 in Appendix S1) than the entire list of transporter genes (Figure S5 in Appendix S1 *versus* Figure 3). The location-dependent differential expression could be explained in terms of sample size (Table S1 in Appendix S1), with more samples available for analysis along the anterior-posterior axis. Only one of the differentially expressed membrane transporter genes (*SLC45A3*) showed a significant, predicted tissue-specific expression pattern using TiGER [23]. Because *SLC45A3* was predicted to show increased expression in prostate tissue it is less relevant to the current study.

**Table 3:** Membrane transporters with differential expression along the anterior-posterior axis.

**Symbol**	Fold Change*	***P*-value**	**FDR**	**Gene Function** [50]
*SLC45A3*	1.9	4.5 × 10^-4^	0.035	Associated with prostate cancer
*SLC46A1*	1.9	2.3 × 10^-5^	0.0094	H^+^-coupled high-affinity folate transporter and intestinal heme transporter
*SLC6A9*	-1.8	8.4 × 10^-5^	0.017	High affinity, Na^+^- and Cl^-^-dependent neurotransmitter transporter
*SLC9A1*	-1.5	3.0 × 10^-4^	0.029	Na^+^/H^+^ antiporter involved in pH regulation to eliminate acids generated by active metabolism or to counter adverse environmental condition
*SLC10A6*	-1.6	4.1 × 10^-5^	0.013	Transports sulfo-conjugated steroid hormones, as well as taurolithocholic acid-3-sulfate and sulfo-conjugated pyrenes in a Na^+^-dependent manner
*SLC45A3*	-1.5	5.6 × 10^-4^	0.039	Associated with prostate cancer

FDR, false discovery rate.

* Positive value indicates up-regulation; negative value indicates down-regulation.

### Microarray gene expression cross-validation by RT-qPCR

Microarray technology has matured significantly over the past decade and now provides a powerful, quantitative tool for gene expression research. The MicroArray Quality Control (MAQC) project (http://www.fda.gov/ScienceResearch/BioinformaticsTools/MicroarrayQualityControlProject/default.htm) has demonstrated that inter- and intra-platform reproducibility, sensitivity, and specificity of microarray data can be obtained provided certain quality control measures are followed [24-28], as they were here. Despite these advances, reverse transcription (RT) analyses with quantitative PCR (qPCR) methods in RT-qPCR assays still are considered the gold standard in quantifying gene expression (i.e., numbers of mRNA transcripts) and relating these measurements to biological activity and ecological function [29].

**Figure 6:**
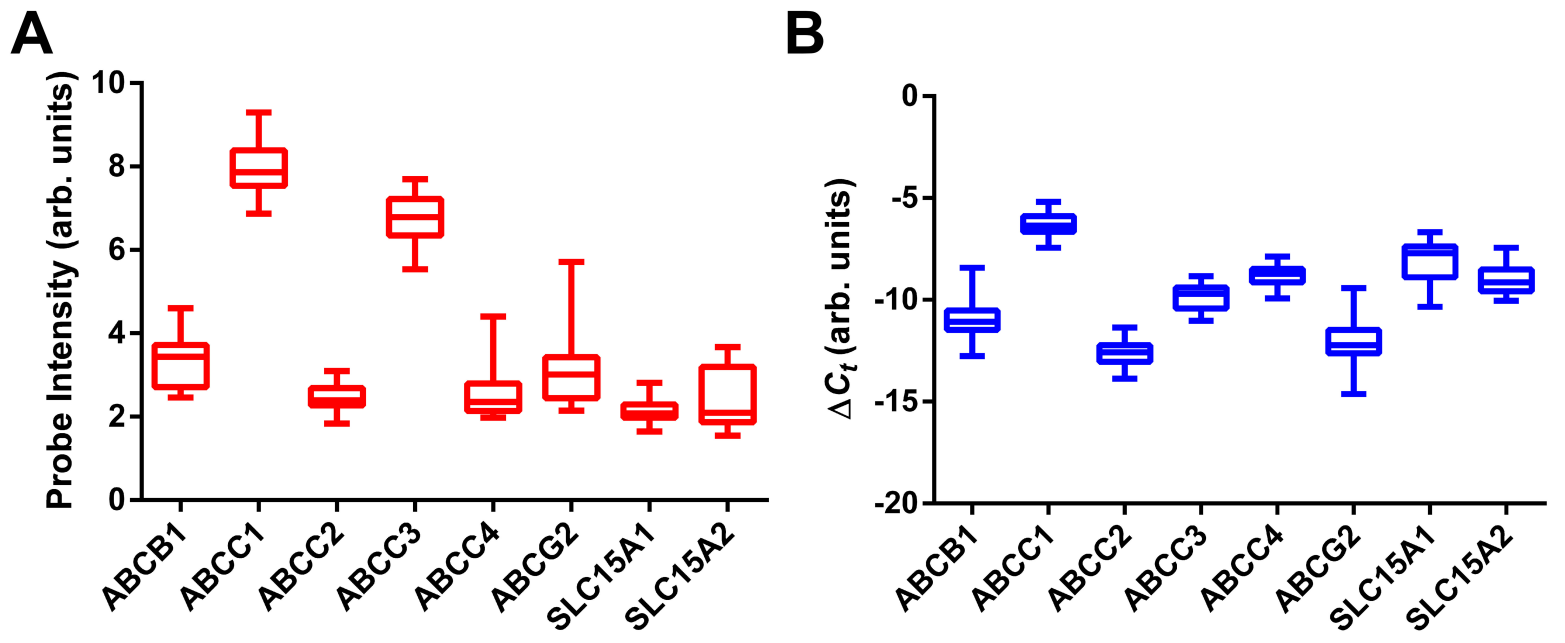
Box plots of expression values for select membrane transporters in 38 vaginal samples from the 6 subjects. The box extends from the 25th to 75th percentiles, with the horizontal line in the box representing the median; whiskers represent the lowest and highest datum. (**A**) Normalized probe intensity data from microarray experiments. (**B**) Normalized RT-qPCR measurements, expressed as 1/[Δ(C_t_)] such that increased values correspond to increased gene expression.

**Figure 7:**
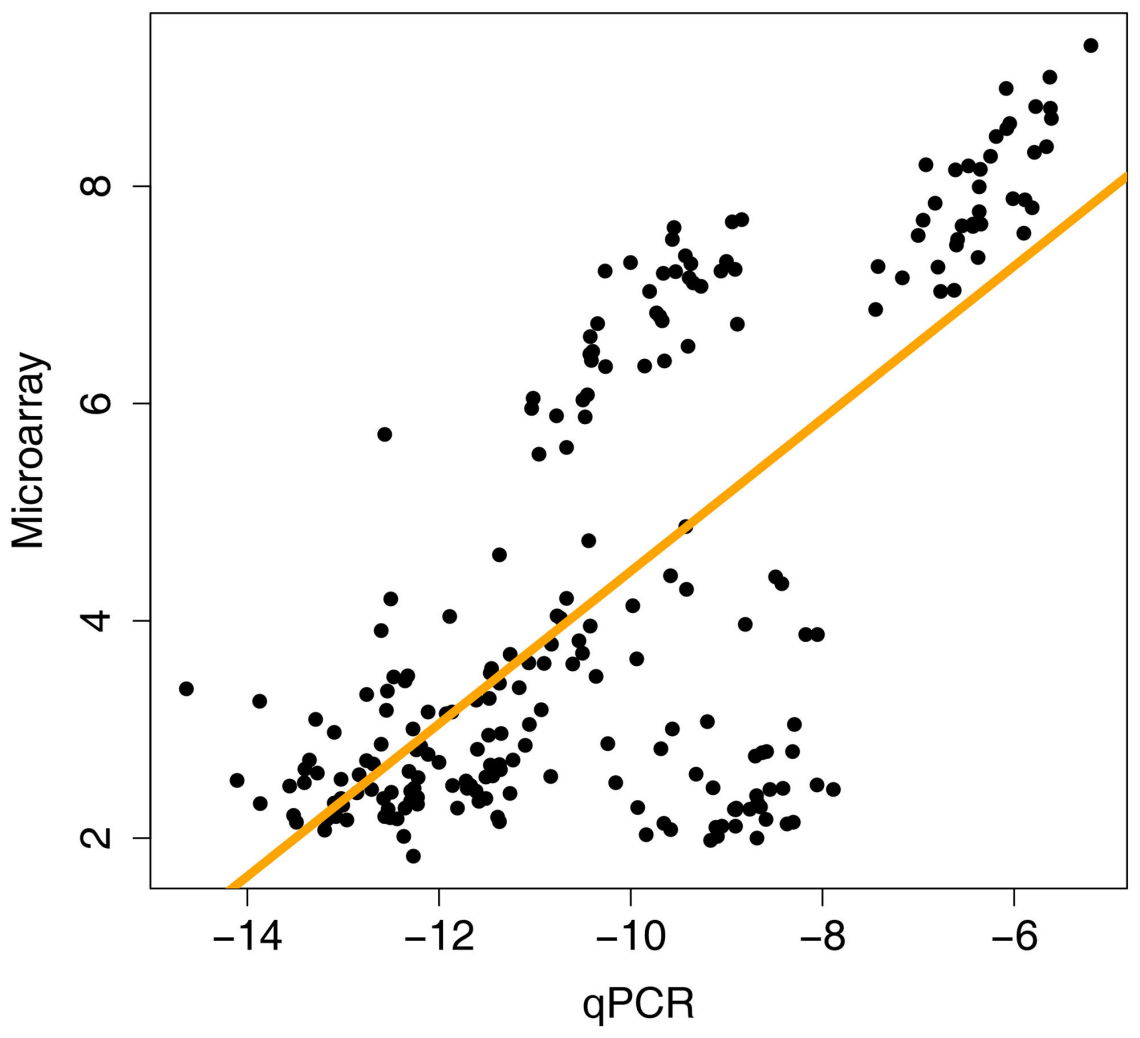
Correlation of gene expression for the six ARV membrane transporter genes (*ABCB1*, *ABCC1*, *ABCC2*, *ABCC3*, *ABCC4*, *ABCG2*) across microarray and qPCR platforms (*R* ^2^ = 0.70, *P* < 2.2×10^-16^).

Ten genes expressing molecular transporters known [13] to affect the disposition of PrECP AVRs and their prodrugs (see Discussion below) –i.e., *ABCB1*, *ABCC1*, *ABCC2*, *ABCC3*, *ABCC4*, *ABCG2*, *SLC15A1*, *SLC15A2*, *SLC22A6*, and *SLC22A8*- were cross-validated with microarray measurements by RT-qPCR analysis using RNA extracted from 41 vaginal tissue samples. The resulting comparison (Figure 6) demonstrated high concordance between these data sets. Note that *SLC22A6* and *SLC22A8* expression are not shown because these transcripts were below the detection threshold of the RT-qPCR assay and also were highly under-expressed in the microarray measurements (median ± standard deviation: *SLC22A6*, 2.11 ± 0.19; *SLC22A8*, 2.04 ± 0.15). The correlation of gene expression for the six ARV membrane transporter genes (*ABCB1*, *ABCC1*, *ABCC2*, *ABCC3*, *ABCC4*, *ABCG2*) across both platforms is shown in Figure 7 (*R* ^2^ = 0.70, *P* < 2.2×10^-16^). *R* ^2^ values as high as 0.82 were observed when the expression values for individual genes were correlated. The cross-platform validation is even more striking when considering that the comparison involved a measure of mRNA transcript abundance, not differentially expressed genes (DEGs) of a study group compared to a control group. The measurements shown in Figure 6 were made using cDNA templates (i.e., reverse transcribed mRNA) prepared in different laboratories from the same pool of RNA. Specific target sequences in these samples were quantified using different molecular probe strategies (i.e., one or more microarray probes *versus* qPCR primer arrays) on two distinct technology platforms, each yielding data that were analyzed by unique computational methods. 

### Immunofluorescence labeling of membrane transporter proteins in vaginal tissues

**Figure 8:**
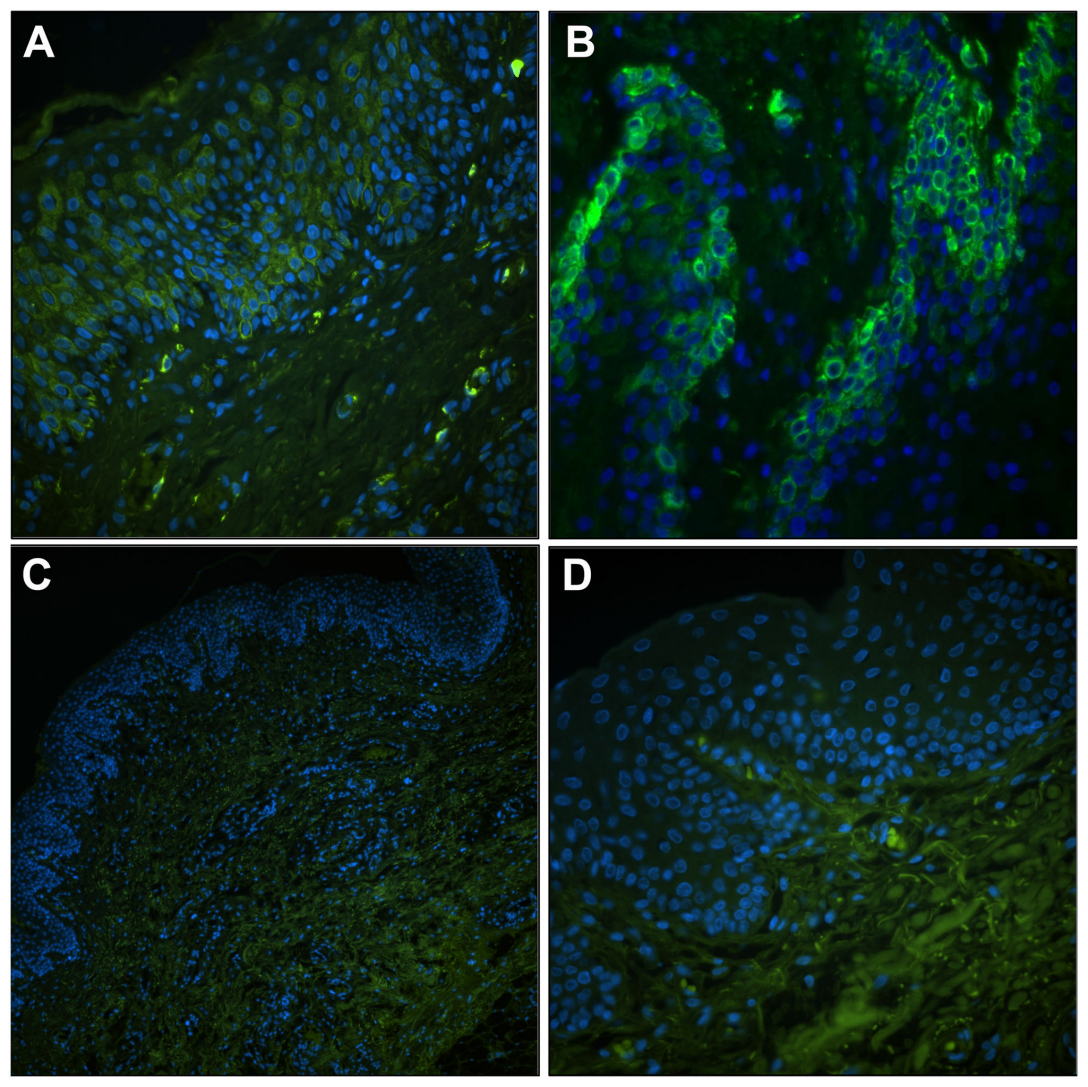
Immunolocalization of MRP3 and MRP4 proteins in vaginal tissue sections. Antibody labeling of target transporter proteins (FITC, green) was completed on at least 5 sections (8 µm) from selected vaginal tissue specimens and at least three fields of labeling were observed. The position of cell nuclei (DAPI, blue) provided architectural cues to orient the tissues. (A) Representation (20×) of MRP3 localization to the surface of epithelial cells (EC). (B) At higher magnification (40×), in some of the tissues, the majority of label was observed in basal, progenitor EC, but the more apical cells also were positive. (C-D) In contrast, MRP4 labeling was localized to the deeper tissue layers and was not found in EC (C, 10×; D, 40×).

At the protein level, expression of the above molecular transporters –i.e., BCRP, MDR-1, MRP-1, MRP-2, MRP-3, MRP-4, OAT-1, and OAT-3- in formalin-fixed vaginal tissues was determined by immunofluorescence. Translation of some of the most highly expressed and biologically relevant transporters was confirmed by immunoblot analysis of tissue lysates. After expression of the 8 selected proteins was confirmed (data not shown), immunefluorescent localization was used to determine the cell types that expressed the proteins (Figure 8). Paraffin sections (8 µm) from full thickness vaginal tissues were processed, and the resulting observations are summarized in Table 4. This initial evaluation indicated cell type-specific expression patterns as shown for MRP3 and MRP4 (Figure 8). The micrographs showed that MRP3 was most highly expressed by epithelial cells (EC) in the vaginal tissue (Figure 8A) with some of the tissues showing localization to the basal, progenitor EC layers. MRP4 was localized to areas that contained fibroblasts, endothelium, and muscle cells (Figure 8C-D). These results confirm the validity of the microarray and qPCR outcomes and indicate the subsequent studies needed to understand the expression patterns of these important host proteins in the vagina.

**Table 4:** Summary of immunolocalization experiments for membrane transporter proteins in vaginal tissue specimens from the 6 subjects spanning multiple locations.

	**Protein (Gene)**								
**Subject ID, Sample Location**	**BCRP (*ABCG2*)**	**MDR1 (*ABCB1*)**	**MRP1 (*ABCC1*)**	**MRP2 (*ABCB2*)**	**MRP3 (*ABCB3*)**	**MRP4 (*ABCB4*)**	**OAT1 (*SLCA6*)**	**OAT3 (*SLCA8*)**	**Sec**
5001, Ant-Int	Musc + wk	-	Endo +	Endo +	EC + str	EC +	Musc/Endo +	Endo +	-
5001, Post-Int	Musc/EC +	-	Endo +	Musc/Endo +	EC + str	-	Musc +	EC +	-
5001, Post-Ext	Musc/Endo +	EC/Progen +	Endo +	Musc/Endo +	EC + str	Musc/Endo +	EC/Progen +	EC/Endo +	-
5002, Post-Int	Musc + wk	-	Endo +	Endo +	EC +	-	Musc/EC +	Endo + wk	-
5003, Post-Int	Musc +	-	Endo +	Ap EC/ Endo +	EC + str	Musc/Endo +	+ wk	EC/Endo + wk	-
5004, Post-Int	Musc +	Fibro/Endo +	Endo +	Endo +	EC + str	Musc/Endo +	Musc + wk	Musc/Endo + wk	-
5005, Post-Int	Musc + wk	Fibro/Endo + wk	-	Musc/Endo +	EC + wk	Musc/Endo +	Musc + wk	Musc + wk	-
5006, Post-Int	Musc +	Fibro/Endo +	-	Musc/Endo +	EC + str	Endo + wk	Musc + wk	Musc + wk	-

Data represent results from at least 5 distinct 8 µm sections and at least 5 low (10×) and high (40×) magnification fields. Tissue collection locations (see Figure 1): Ant, anterior; Post, posterior; Int, interior; Ext, exterior. Cell types where membrane transporters were localized: Musc, muscular tissue; EC, epithelial cells; Endo, endothelial cells; Progen, progenitor epithelial cells; Fibro, fibroblast; + membrane transporter protein consistently observed by immunofluorescence microscopy; - membrane transporter protein not observed by immunofluorescence microscopy; str, strong signal/expression; wk, weak signal/expression.

## Discussion

The vaginal mucosal surface consists of a stratified squamous epithelium resting on an indistinct lamina propria and an underlying vascular submucosa [30]. This multi-layered structure can be a few to 45 cells thick and is made up of four zones [31]. The unique anatomy, physiology, microbiome, and function of the VT suggest that the vaginal tissues may have evolved to express a unique suite of molecular transporters to support these functions. Prior to our study virtually no data were available on the distribution of molecular transporters in these tissues. Kuchiiwa et al. found intense expression of MCT1 (*SLC16A1* gene product), a monocarboxylate transporter, with concomitant expression of GLUT1 (*SLC2A1* gene product), a glucose transporter, in the vaginal epithelium of mice when studying genital lactate shuttling [22], results in close agreement to our expression measurements in the human VT (Table 2).

Using whole-genome transcriptome analysis, we have shown that vaginal tissues in six women with highly diverse characteristics (Table 1) consistently overexpress a 235 membrane transporter gene isoforms (43% of the known transporter genes). This unexpected finding suggests that, like the small intestine [32], the VT has a broad functional diversity for absorptive and secretory carrier-mediated transport of xenobiotics. Studies involving drug disposition, including drug-drug interactions, following oral or intravaginal administration may need to include a microarray component to faithfully capture the subtleties of membrane transporter expression. Shotgun mass spectrometry proteomics [33] could be highly complementary to these studies by allowing the complete membrane proteome to be analyzed in tandem with the genome-wide transcriptome. The large number of potentially relevant expressed genes most likely render qPCR and immunolabeling impractical at this stage, but may be useful in future studies when a key subset of relevant transporters has been identified. 

The vaginal mucosal epithelium is more than a passive physical barrier protecting from infection. It functions as an active front line of the host immune system [34]. During sexual transmission, HIV enters the cervicovaginal mucosa and can cross the epithelial barrier within hours [35] to establish a small founder population of infected cells [36,37]. Effective HIV PrECP using ARV drugs is believed to involve the eradication of this founder population so that it cannot undergo expansion and establish a self-propagating systemic infection throughout the secondary lymphoid organs [37,38]. For this strategy to be successful, ARV drugs must be present at sufficiently high levels in the key anatomic compartments for pharmacologic activity. In the case of female-controlled, heterosexual HIV PrECP, these compartments involve the vaginal tissues, with specific target anatomic sites depending on the ARV drug mechanistic class [13]. Kinetic interactions between ARV drugs and membrane transporters in the VT therefore will play a fundamentally important role in determining the pharmacodynamic (PD) outcomes.

The role of ABC and SLC transporters in ARV therapy has been discussed in a recent review and key absorptive/secretory pathways in various anatomic compartments, excluding the VT, were identified [13]. While a detailed discussion on the impact of molecular transporters in the VT on ARV drug disposition is beyond the scope of this report, some preliminary inferences can be made. The three FDA-approved ARVs currently being evaluated for PrECP include the nucleoside reverse transcriptase inhibitors (NRTIs) tenofovir (TFV) and emtricitabine (FTC) and the CCR5 antagonist maraviroc (MVC). Vaginal gels delivering these drugs either individually or in combination have demonstrated efficacy in the macaque model [39,40]. A TFV vaginal gel and an oral formulation of tenofovir disoproxil fumarate (TDF, prodrug of TFV) and FTC are under clinical evaluation for PrECP [11]. Interestingly, all three ARVs show little affinity for transporters from the SLC superfamily, with TFV [41,42] and FTC [43] acting as substrates for OAT1 (*SLC22A6* gene product) and OAT3 (*SLC22A8* gene product). Microarray and RT-qPCR measurements found SLC22A6- and SLC22A6-transcript expression to be low in vaginal tissues. These findings were supported by immunolocalization of the corresponding proteins, which were present at low abundance in the endothelium and underlying muscular tissue (Table 4). Another member of this transporter family (*SLC22A18*) was highly expressed in all vaginal tissue samples analyzed here (Table 2), and nine members of the SLC22 family were over-expressed across all samples (Dataset S1). 

Transporters from the ABC superfamily play a more prominent role in the disposition of TFV, FTC, and MVC. All three are either substrates for or inhibit MDR1 (P-gp), the *ABCB1*-gene product [13], which was under-expressed in all samples (Figure 6, Dataset S1). MDR1 immunolabeling measurements supported these findings and only were able to detect low abundance of this protein, associated primarily with fibroblast and endothelium, in ca. half the samples analyzed (Table 4). FTC and TFV are either substrates or inhibitors of members of the ABCC family, notably MRP1-4 (*ABCC1-4*) [13]. *ABCC1* and *ABCC3* were over-expressed in all samples (Figure 6). MRP1 was associated primarily with endothelial cells, while MRP3 was expressed predominantly by epithelial cells (Figure 8, Table 4). These findings suggest that these efflux systems could play competing roles in TFV/FTC excretion from cells in different strata of vaginal tissues. TFV and FTC also are substrates for BCRP (*ABCG2*-gene product) [13,43], which was under-expressed across all samples (Figure 6).

In conclusion, molecular transporters important to the disposition of TFV, FTC, and MVC were consistently over- or under-expressed in all vaginal tissue samples. Different vaginal tissues and cell types likely will exhibit different cellular pharmacology of these ARV drugs as a result of heterogeneous transporter expression. While these molecular transporters form promising targets for future qPCR measurements in clinical studies involving subjects receiving one or more ARV drugs for HIV PrECP, they should be supported by global microarray analysis of the membrane transporter transcriptome to ensure that no important interactions are missed. Protein visualization in specific tissue/cell types also forms an important complementary analysis, as shown here, once the relevant genes have been identified.

Prodrugs of ARV agents can increase the bioavailability of the parent moiety for topical delivery to the VT. For example, we showed that intravaginal rings (IVRs) delivering TFV and TDF at the same rate to the vaginal lumen *in vivo* resulted in dramatically different TFV tissue levels, with the TDF IVRs affording levels nearly two orders of magnitude greater than the TFV IVRs [44]. Targeting molecular transporters in the VT for enhanced bioavailability may provide a powerful strategy in the rational design and selection of ARV prodrugs in topical HIV PrECP. Existing antiviral prodrugs that benefit from carrier-mediated transport to increase their oral bioavailability include the commercially available *L*-valyl ester of acyclovir (valacyclovir) and mono- and di-peptide prodrugs of acyclovir, ganciclovir, and saquinavir developed by Mitra and colleagues [45-48]. All these prodrugs target PEPT1 (*SLC15A1*-gene product) and, to a lesser extent, PEPT2 (*SLC15A2*-gene product), membrane transporters that were under-expressed in all samples measured here (Figure 6). Our findings suggest that the peptidic prodrugs may not significantly enhance the bioavailability of the parent compound if administered intravaginally, although additional research is required to more thoroughly characterize these interactions. Given the successful evaluation reported in this global analysis of the VT transcriptome, the most highly expressed of molecular transporters present in the VT can now be better targeted with refined drug design.

## Supporting Information

Appendix S1 Supporting figures and tables.
Figure S1. Clustering of samples analyzed by Principal Component Analysis (PCA). Figure S2. Dendrogram showing hierarchical clustering of vaginal samples. Figure S3. Distribution of averaged expression patterns for membrane transporters. Figure S4. Example of a transporter gene SLC44A1 with universally high expression patterns in the TiGER database. Figure S5. Hierarchical clustering of membrane transporter expression across the anterior-posterior axis of the vaginal tract. Figure S6. Sources of variation in global gene expression. Figure S7. Sample histogram. Table S1. Summary of 41 vaginal tissue sample number per location for each subject used in the differential gene expression analyses. Table S2. Tissue-specific scores for highly expressed membrane transporters.
(PDF)

Dataset S1 **Microarray membrane transporter gene expression dataset.**
(XLS)
