# Supplementary material for: Global Expression of Molecular Transporters in the Human Vaginal Tract: Implications for HIV Chemoprophylaxis
Source: PLoS One. 2013 Oct 15;8(10):e77340. doi: 10.1371/journal.pone.0077340 (PMC3797116; doi:10.1371/journal.pone.0077340)
Supplement: Appendix S1 — Supporting figures and tables. Figure S1. Clustering of samples analyzed by Principal Component Analysis (PCA). Figure S2. Dendrogram showing hierarchical clustering of vaginal samples. Figure S3. Distribution of averaged expression patterns for membrane transporters. Figure S4. Example of a transporter gene SLC44A1 with universally high expression patterns in the TiGER database. Figure S5. Hierarchical clustering of membrane transporter expression across the anterior-posterior axis of the vaginal tract. Figure S6. Sources of variation in global gene expression. Figure S7. Sample histogram. Table S1. Summary of 41 vaginal tissue sample number per location for each subject used in the differential gene expression analyses. Table S2. Tissue-specific scores for highly expressed membrane transporters. (PDF) [file pone.0077340.s001.pdf]

Global Expression of Molecular Transporters in the Human Vaginal Tract: Implications for HIV Chemoprophylaxis

Manjula Gunawardana[1], Madeline Mullen[1], John A. Moss[1], Richard B. Pyles[2], Rebecca J. Nusbaum[3], Jignesh Patel[2], Kathleen L. Vincent[4], Charles Wang[5], Chao Guo[5], Yate-Ching Yuan[6], Charles D. Warden[6], Marc M. Baum[1*]

[1]Department of Chemistry, Oak Crest Institute of Science, 2275 E. Foothill Blvd., Pasadena, CA 91107, USA, [2]Departments of Pediatrics and Microbiology and Immunology, UTMB. 301 University Blvd, Galveston, TX 77555, USA, [3]Human Pathophysiology and Translational Medicine Graduate Program, UTMB. 301 University Blvd, Galveston, TX 77555, USA, [4]Center for Biomedical Engineering, 301 University Boulevard, University of Texas Medical Branch at Galveston, TX 77555, USA, [5]Functional Genomics Core, Beckman Research Institute, City of Hope Comprehensive Cancer Center, 1710 Flower Ave., Duarte, CA 91010, USA, [6]Bioinformatics Core, Department of Molecular Medicine, City of Hope National Medical Center, 1500 East Duarte Rd., Duarte, CA 91010, USA.

Supplementary Materials

Fig. S1. Clustering of samples analyzed by Principal Component Analysis (PCA). Samples cluster primarily by subject, and no strong outliers are apparent. Subject ID: 1, red; 2, dark blue; 3, green; 4, purple; 5, orange; and 6, cyan.

Fig. S2. Dendrogram showing hierarchical clustering of vaginal samples. (**A**) Color bar indicates strong clustering among samples from the same subject, for subjects 1-6. Color bars defining clustering along the following axes in the vaginal tract (Fig. 1) show no discernible pattern: (**B**) anterior-posterior, (**C**) distal-proximal, and (**D**) left-right.

Fig. S3. **Distribution of averaged expression patterns for membrane transporters**. Average expression values per probe across samples (N = 44), sorted in descending order.

Fig. S4. **Example of a transporter gene SLC44A1 with universally high expression patterns in the TiGER database**. Notice practically all of this tissues show some level of enrichment and there are no tissue-specific signatures (with enrichment score stronger than 5 for a particular tissue).

Fig. S5. **Hierarchical clustering of membrane transporter expression across the anterior-posterior axis of the vaginal tract**. (A) Color bars show increased clustering pattern along the anterior-posterior axis in the vaginal tract (Fig. 1), when compared to the clustering observed for all membrane transporters (Fig. 3 and Fig. S2). The heatmap shows clustering of the standardized expression values, where probes with high expression are shown in red and probes with low expression are shown in blue (see color key below the heatmap). *SLC46A1* and *SLC45A3* appear in a cluster that is up-regulated in anterior samples, while *SLC35F2*, *SLC9A1*, *SLC6A9*, and *SLC10A6* appear in a cluster that is up-regulated in posterior samples.

Fig. S6. Sources of variation in global gene expression. A-P, anterior-posterior axis; P-D, proximal-distal axis; and L-R, left-right. ANOVA was used to compare the above factors and F-statistics were calculated and averaged across all genes on the microarray. Average F-statistics for each variable were divided by the average F-statistic for unexplained variability (Error), and these ratios are shown in the figure. For example, the ratio for L-R is 1.18, meaning that this variable explained 18% more variation than would be explained by a 5th variable associated with

random noise. Likewise, the subject ID can explain approximately twice the variation observed for A-P.

Fig. S7. **Sample histogram**. An over-expression threshold of 3.5 was determined from this plot.

Table S1. Summary of 41 vaginal tissue sample number per location for each subject used in the differential gene expression analyses. The sample location in the vaginal tract is shown schematically in Fig. 1.

Table S2. Tissue-specific scores for highly expressed membrane transporters. Tissue-specific expression requires a score > 5 and *P*-value < 10-3.5. N/A, gene not present in database.

Dataset S1. Over-expression and averaged expression data in tabular form for all molecular transporters.

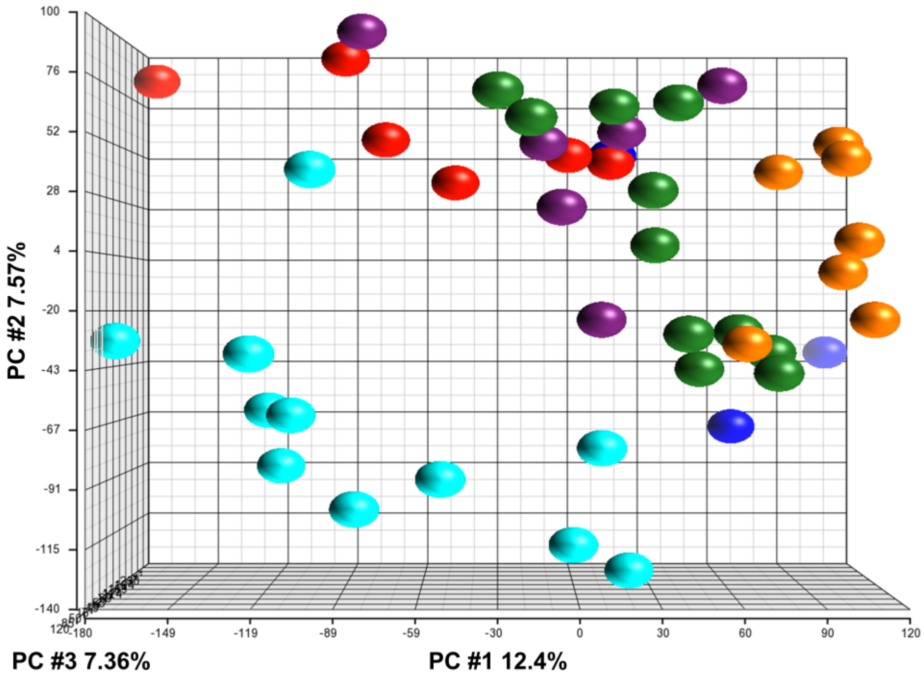

Fig. S1. Clustering of samples analyzed by Principal Component Analysis (PCA). Samples cluster primarily by subject, and no strong outliers are apparent. Subject ID: 1, red; 2, dark blue; 3, green; 4, purple; 5, orange; and 6, cyan.

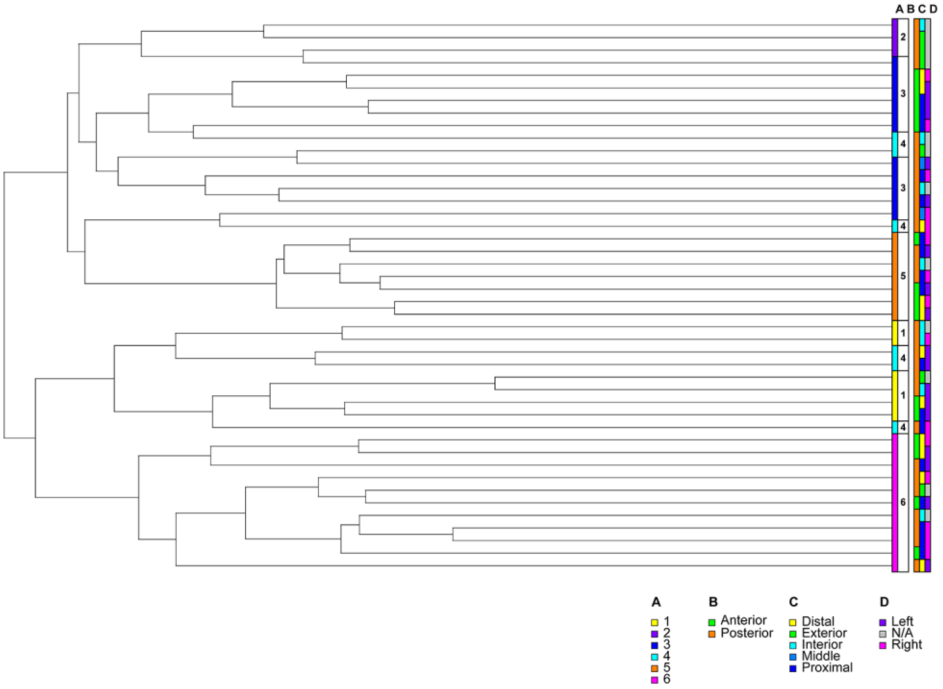

Fig. S2. Dendrogram showing hierarchical clustering of vaginal samples. (**A**) Color bar indicates strong clustering among samples from the same subject, for subjects 1-6. Color bars defining clustering along the following axes in the vaginal tract (Fig. 1) show no discernible pattern: (**B**) anterior-posterior, (**C**) distal-proximal, and (**D**) left-right.

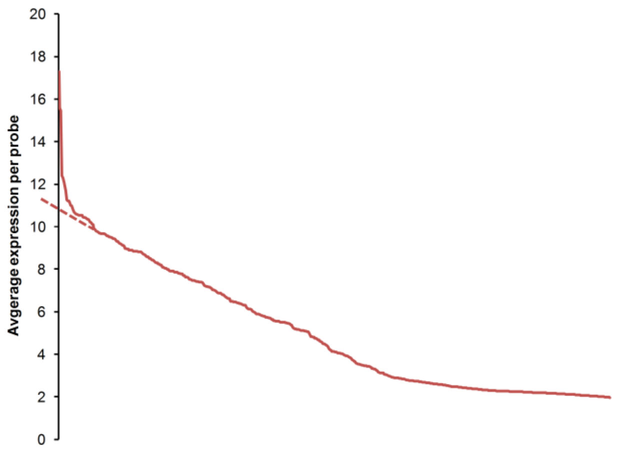

Fig. S3. Distribution of averaged expression patterns for membrane transporters. Average expression values per probe across samples (*N* = 44), sorted in descending order.

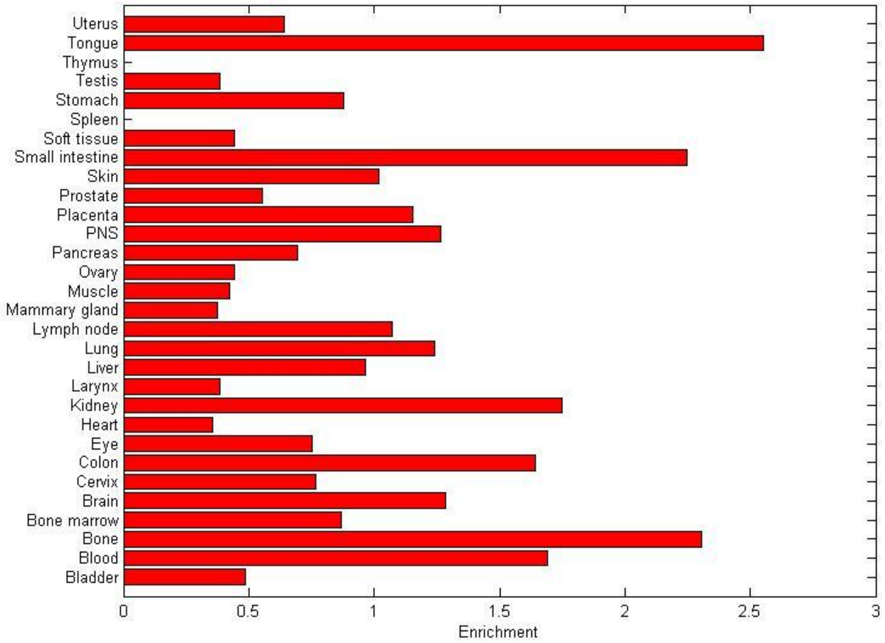

Fig. S4. **Example of a transporter gene *SLC44A1* with universally high expression patterns in the TiGER database**. Notice practically all of this tissues show some level of enrichment and there are no tissue-specific signatures (with enrichment score stronger than 5 for a particular tissue).

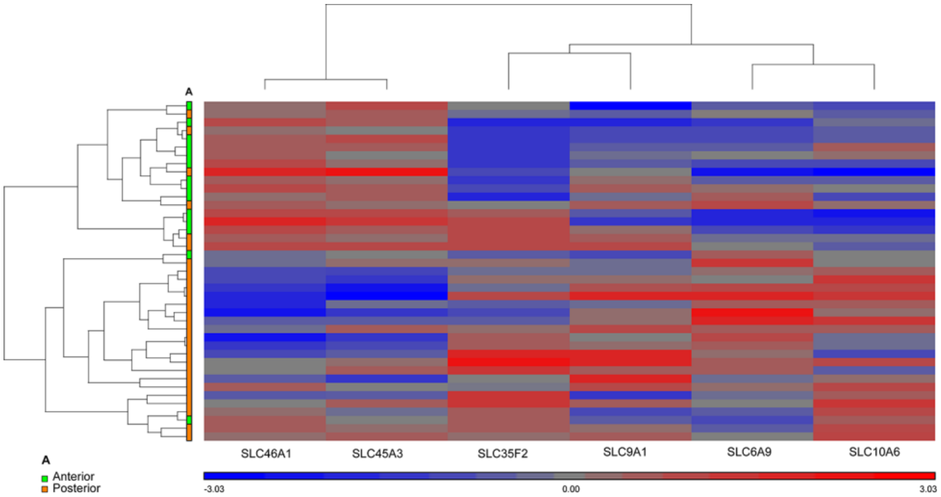

Fig. S5. **Hierarchical clustering of membrane transporter expression across the anterior-posterior axis of the vaginal tract**. (A) Color bars show increased clustering pattern along the anterior-posterior axis in the vaginal tract (Fig. 1), when compared to the clustering observed for all membrane transporters (Fig. 3 and Fig. S2). The heatmap shows clustering of the standardized expression values, where probes with high expression are shown in red and probes with low expression are shown in blue (see color key below the heatmap). *SLC46A1* and *SLC45A3* appear in a cluster that is up-regulated in anterior samples, while *SLC35F2*, *SLC9A1*, *SLC6A9*, and *SLC10A6* appear in a cluster that is up-regulated in posterior samples.

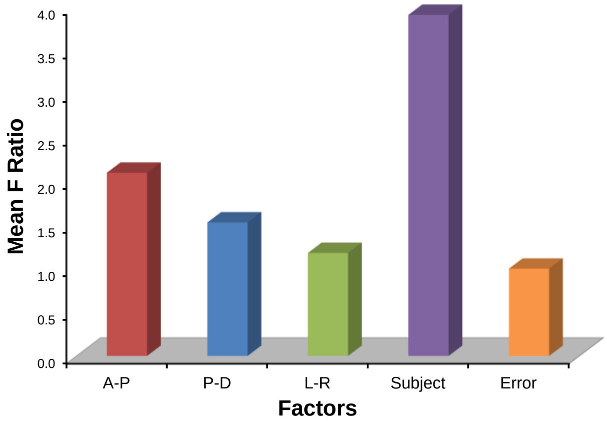

Fig. S6. **Sources of variation in global gene expression**. A-P, anterior-posterior axis; P-D, proximal-distal axis; and L-R, left-right. ANOVA was used to compare the above factors and F-statistics were calculated and averaged across all genes on the microarray. Average F-statistics for each variable were divided by the average F-statistic for unexplained variability (Error), and these ratios are shown in the figure. For example, the ratio for L-R is 1.18, meaning that this variable explained 18% more variation than would be explained by a 5th variable associated with random noise. Likewise, the subject ID can explain approximately twice the variation observed for A-P.

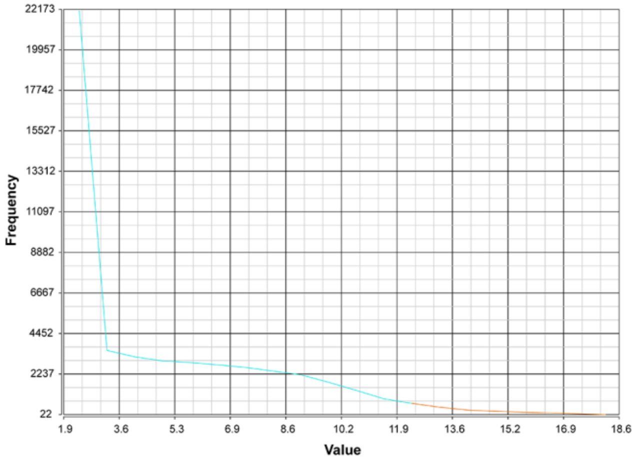

Fig. S7. **Sample histogram**. An over-expression threshold of 3.5 was determined from this plot.

Table S1. Summary of 41 vaginal tissue sample number per location for each subject used in the differential gene expression analyses. The sample location in the vaginal tract is shown schematically in Fig. 1.

	**Subject ID**					
**Sample Location***	**1**	**2**	**3**	**4**	**5**	**6**
Anterior, proximal			2		2	2
Anterior, distal	2		2		2	2
Posterior, proximal			2	2	2	2
Posterior, distal			2†	2		2
Posterior, interior	3	1	1	1	1	1
Posterior, exterior	1	1	1	1		1

*Usually collected from right and left side of vaginal wall.

†Identified as "posterior middle".

Table S2. Tissue-specific scores for highly expressed membrane transporters. Tissue-specific expression requires a score > 5 and *P*-value < 10-3.5. N/A, gene not present in database.

Gene Symbol	Tissue-specific Enrichment	Uterus Score	Ovary Score	Cervix Score
RHCG	larynx, tongue	0.24	0	1.0
TAP1	none	3.0	0.77	3.1
SLC2A1	none	0.46	1.7	0.58
SLC6A9	none	0	0	4.1
SLC16A3	none	0.48	2.3	2.3
SLC16A12	kidney, pancreas	0.94	0	0
SLC22A18	colon	0	0.42	0.98
SLC24A3	none	1.8	0	0
SLC35E1	none	1.4	1.3	0.84
SLC35E4	kidney, pancreas	0.94	0	0
SLC44A1	none	0.64	0.44	0.77
SLC38A10	N/A	N/A	N/A	N/A
